# Low maternal education increases the risk of Type 1 Diabetes, but not other autoimmune diseases: a mediating role of childhood BMI and exposure to serious life events

**DOI:** 10.1038/s41598-023-32869-x

**Published:** 2023-04-15

**Authors:** Pär Andersson White, Tomas Faresjö, Michael P. Jones, Johnny Ludvigsson

**Affiliations:** 1 Department of Health, Medicine and Care, General Practice, Linköping University, Linköping, 581 83 Sweden; 2 Crown Princess Victoria Children’s Hospital, Linköping University, Linköping, 581 85 Sweden; 3 School of Psychological Sciences, Macquarie University, North Ryde, NSW 2109 Australia; 4 Department of Biomedical and Clinical Sciences, Division of Pediatrics, Linköping University, Linköping, 581 83 Sweden

**Keywords:** Type 1 diabetes, Epidemiology, Epidemiology, Autoimmune diseases, Risk factors

## Abstract

The objective of this paper was to investigate if socioeconomic status (SES), measured by maternal education and household income, influenced the risk of developing autoimmune disease (Type 1 Diabetes, Celiac disease, Juvenile Idiopathic Arthritis, Crohn’s disease, Ulcerative colitis, and autoimmune thyroid disease), or age at diagnosis, and to analyse pathways between SES and autoimmune disease. We used data from the All Babies in Southeast Sweden (ABIS) study, a population-based prospective birth cohort, which included children born 1997–1999. Diagnoses of autoimmune disease was collected from the Swedish National Patient Register Dec 2020. In 16,365 individuals, low maternal education, but not household income, was associated with increased risk of Type 1 Diabetes; middle education RR 1.54, 95% CI 1.06, 2.23; P 0.02, low education RR 1.81, 95% CI 1.04, 3.18; P 0.04. Maternal education and household income was not associated with any other autoimmune disease and did not influence the age at diagnosis. Part of the increased risk of Type 1 Diabetes by lower maternal education was mediated by the indirect pathway of higher BMI and higher risk of Serious Life Events (SLE) at 5 years of age. The risk of developing Type 1 Diabetes associated to low maternal education might be reduced by decreasing BMI and SLE during childhood.

## Introduction

During the last decades the incidence of the major pediatric chronic autoimmune diseases has increased rapidly^1^. Type 1 Diabetes has had an estimated annual increase of incidence of 2.4% per year worldwide, and 3–4% in Europe^2,3^. The incidence of Celiac disease have risen remarkably both in Sweden and the United Kingdom^4,5^ and the prevalence of inflammatory bowel diseases (IBD), which includes Crohn’s Disease and Ulcerative Colitis follows the same pattern^6^. The evidence for an increase in Autoimmune thyroid disease is less clear, but there are some evidence of a similar development as for other autoimmune diseases^7^. The development the incidence of Juvenile Idiopathic Arthritis (JIA) remains unclear due to lack of long prospective studies and varying diagnostic criteria over the years and in different regions^8^.

Genetics, especially HLA-type, plays a major role in the risk of developing autoimmune disease^9,10^. However, the rapid rise of disease incidence cannot be explained by changes in our genes. Rather an explanation to this phenomenon must be sought in our environment or lifestyle^11^. Some hypotheses have been suggested based on environmental factors that have changed over the last decades, including the hygiene hypothesis, viral infections, dietary changes, changes in our gut microbiota, lack of vitamin D and psychosocial stress^12,13^.

These environmental factors that increase the risk of autoimmune disease might be unequally distributed in the population and give rise to socioeconomic status (SES) differences. SES, which can be measured by income, educational attainment, or occupation, has been repeatedly observed to have an inverse relationship with the risk of several health problems and diseases, including cardiovascular disease, with the risk of these diseases being higher with each step down the SES ladder^14,15^. The data remain inconclusive on whether autoimmune diseases follow this pattern and have a SES gradient^16^. As an example some studies on Type 1 Diabetes have indicated increased risk among families with low SES^17^. However, other studies show no differences or a decreased risk^18,19^.

The pathways between SES and disease are complex. The increased risk in low SES population is often mediated by other behavior and risk factors i.e., SES increases the risk of smoking and obesity which in turn increases the risk of cardiovascular disease^20^. Such mediating factors will differ between different diseases/outcomes. For a variable to be a mediator between SES and an autoimmune disease it must be both associated with higher risk of that specific disease and be associated with SES. There is reason to believe that Type 1 Diabetes could potentially have unique pathways that differ to other autoimmune diseases, as findings indicate that Type 1 Diabetes development is influenced not only by the autoimmune process but also with increased demand for insulin for example due to higher BMI or psychological stress^21,22^.

The first aim of this study was to investigate whether SES was associated with the risk of developing autoimmune diseases. A second aim was to investigate whether SES influenced the age at diagnosis of autoimmune disease. A third and final aim was to analyse, in diseases where a SES association could be established, pathways between SES and disease. Our hypothesis was that the increased health inequality seen for many diseases including cardiovascular disease would be reflected also in autoimmune diseases, with a gradient between low and high SES groups.

## Methods

### Study participants

The All Babies In Southeast Sweden (ABIS) study is a prospective population based cohort study where all families with children born between the 1 October 1997 and 1 October 1999 in southeast Sweden were invited to participate. 17,055 children participated out of approximately 21,600 children (78.6%) who were born in the area during the inclusion period. Questionnaires as well as biological samples were collected at regular follow-ups.

The parents were given oral and written information before giving informed consent to participate in the ABIS study. The ABIS study was approved by the research ethics committees at Linköping University (Dnr 96-287, Dnr 99-321 and Dnr 03-092) and Lund University (LU 83-97) in Sweden, and connection of the ABIS registers to National registers was approved by the Research Ethics committee in Linköping ‘Dnr2013/253-32. All methods were carried out in accordance with relevant guidelines and regulations.

### Diagnosis of auto-immune diseases

Diagnoses of autoimmune disease were collected by cross-linking with the Swedish National Patient Register, last available date of diagnosis was 2020-12-31. This register collects data on a national level of medical diagnoses with dates for each in and out-patient visit. A search in the register was performed of diagnose codes according to ICD-10. The codes used were E10 for Type 1 Diabetes, K900 for Celiac disease, K50 for Crohn’s disease, K51 for Ulcerative colitis, M08 for Juvenile Idiopathic Arthritis (JIA), and for autoimmune thyroid disease two codes were combined E05 thyrotoxicosis (Graves’ disease) and E063 autoimmune thyroiditis (Hashimoto thyroiditis). Data on medical diagnoses was available for 16 365 participants*.*

### Socioeconomic status

Mothers answered a question about their educational attainment in the first ABIS birth questionnaire. Maternal education was classified according to the International Standard Classification of Education (ISCED) and graded in three levels: low maternal education = ISCED level I–II, middle maternal education = ISCED level III–IV, and high maternal education = ISCED level V–VII^23^.

Household income was collected from the Swedish Income and Tax register for the year 2000. Participants were classified into three groups according to their household income; low income was defined as the bottom income quintile, middle income as the second to fourth quintile and high income as the top income quintile.

### Potential confounder of autoimmune risk

Potential confounders of autoimmunity were reported in the birth questionnaire and included child’s sex, ethnicity dichotomized as both parents born in Sweden or at least one parent born in another country, and heredity which was dichotomized as no heredity or at least one first degree relative with autoimmune disease^24^.

### Potential mediators of SES: autoimmune disease association

The ABIS study contains questionnaire-based data on several potential mediators (i.e., infections during pregnancy, maternal overweight/obesity, child’s BMI, Serious Life Events) between SES and autoimmune disease. Complete information on potential mediators at age 5 was available for N 6782. As a mediation analysis requires exclusion of Type 1 Diabetes cases occurring before the exposure (to avoid implying reverse causality), data from the 5 year questionnaire was chosen in favor of later follow-ups due to having the highest validity in support of causal conclusions. Variables were chosen based on previous research showing an association with autoimmune disease.

BMI was calculated using height and weight reported by parents in questionnaires. The definition of Serious life events was a dichotomous variable defined by answering yes to any of the questions regarding serious life events, the definition has been described in previous publications^21^. In brief, parent answered a question ‘Has your child been exposed to something which you perceive as a serious life event? Yes or No, followed by a list of events that included parental divorce, new family member (adult or non-biological sibling), severe disease or death in the family, unemployment in the family, conflicts between parents, interventions by the social services and exposure to violence. Regarding infections mothers answered a question regarding infections during pregnancy in the inclusion questionnaire at child’s birth.

### Statistics

RRs were estimated using a generalized linear model with a log link and robust variance estimation with confounders entered as covariates in multivariate regressions. Income and education were analysed separately in the multivariate analysis. In analysis of specific autoimmune disease heredity of that disease was adjusted for (i.e., heredity for Type 1 Diabetes in the analysis on Type 1 Diabetes). In analyses of ‘Any autoimmune disease’, heredity for any of the included autoimmune diseases was used. Time to Diagnosis was analysed using zero-inflated negative binominal regression.

The potential role of body mass index (BMI) and serious life events (SLE) in the association between maternal education and the incidence of Type 1 Diabetes was evaluated through path modelling. Maternal education was the primary independent variable (IV), Type 1 Diabetes the dependent variable (DV) and BMI and SLE were included as intermediate variables between IV and DV. Both estimates of parameters representing the direct association between IV and DV as well as the indirect association via BMI and SLE were calculated. The indirect association is reported along with 95% confidence interval and expressed as a fraction of the total association. Formal statistical inference was via the nonparametric bootstrap using 1000 bootstrap samples. All parameter estimates are reported in standardized form.

## Results

The prevalence of autoimmune disease in our study was 3.8%. The most common specific disease was Celiac disease (1.4%), followed by Type 1 Diabetes (1%), JIA (0.5%), Ulcerous colitis (0.5%), Crohn’s disease (0.3%), and Autoimmune thyroid disease (0.3%), see Table 1.

**Table 1:** Characteristics and cumulative incidence of autoimmune disease from birth until age 22 (SD 0.56). N = 16,365.

	N	%
Prevalence of autoimmune diseases		
Type 1 Diabetes	167	1.0
Celiac disease	228	1.4
JIA	86	0.5
Ulcerous colitis	81	0.5
Crohn’s disease	59	0.3
Autoimmune thyroid disease	56	0.3
Any autoimmune disease	630	3.8
Missing	0	0
Sex		
Men	8485	51.8
Women	7880	48.2
Missing	0	0
Ethnicity		
Swedish	14,080	86.0
Other country	1695	10.4
Missing	590	3.6
Maternal educational		
High (ISCED V–VII)	5068	31.0
Middle (ISCED III–IV)	9525	58.2
Low (ISCED I–II)	1379	8.4
Missing	393	2.4
Household income group		
High (5th quintile)	3250	19.9
Middle (2-4th quintile)	9752	59.6
Low (1st quintile)	3250	19.9
Missing	113	0.7
Heredity (first-degree relative)		
Type 1 Diabetes	398	2.4
Celiac disease	223	1.4
JIA/RA	248	1.5
IBD	233	1.4
Autoimmune thyroid disease	405	2.5
Any autoimmune disease	1372	8.4
No heredity	14,773	90.3
Missing	220	1.3

*IBD* Inflammatory Bowel Disease, *ISCED* International Standard Classification of Education, *JIA* Juvenile Idiopathic Arthritis, *RA* Rheumatoid arthritis.

There were differences between sexes; females were more likely to develop an autoimmune disease RR 1.48, 95% CI 1.27, 1.74; P < 0.01. This was due to higher risk of Celiac disease, JIA, and Autoimmune thyroid disease. Non-Swedish ethnicity was associated with a reduced risk of any autoimmune disease, RR 0.56 95% CI 0.40, 0.78; P < 0.01. On an individual disease level, RRs of children to immigrant parents were statistically significantly lower for Celiac disease and Type 1 Diabetes, see Supplementary Table 1.

In multivariate analysis which adjusts for potential confounders (child’s sex, ethnicity, and heredity of autoimmune disease) there was an SES gradient in the risk of Type 1 Diabetes when SES was measured by maternal education; middle education RR 1.54, 95% CI 1.06, 2.23; P 0.02, low education RR 1.81, 95% CI 1.04, 3.18; P 0.04, see Table 2. No other autoimmune disease had a statistically significant association with maternal education. Income was not associated with increased risk in any autoimmune disease. The risk of the combined variable “Any autoimmune disease” was also increased with lower maternal education; middle education RR 1.21, 95% CI 1.01, 1.44; P = 0.04, low education RR 1.29, 95% CI 0.96, 1.73; P = 0.09, but not if Type 1 Diabetes was excluded; RR 1.14, 95% CI 0.81, 1.60; P = 0.46, low education RR 1.11, 95% CI 0.91, 1.35; P = 0.32.

**Table 2:** Association between SES and the relative risk of autoimmune diseases.

	Relative risk of disease			Time to disease diagnosis				
	RR	CI	P	b	SE	Lower	Upper	P
Type 1 diabetes								
Low maternal education	1. 81	(1.04, 3.18)	0.04	0.11	0.16	− 0.21	0.43	0.49
Middle maternal education	1. 54	(1.06, 2.23)	0.02	− 0.03	0.11	− 0.24	0.19	0.81
Low income	0.96	(0.58, 1.59)	0.89	0.11	0.15	− 0.18	0.41	0.45
Middle income	1.07	(0.72, 1.59)	0.73	0.12	0.12	− 0.11	0.35	0.31
Celiac disease								
Low maternal education	0.99	(0.58, 1.68)	0.96	− 0.22	0.17	− 0.55	0.11	0.19
Middle maternal education	1.10	(0.83, 1.48)	0.50	0.05	0.09	− 0.13	0.23	0.59
Low income	0.84	(0.53, 1.34)	0.47	0.00	0.14	− 0.28	0.29	0.98
Middle income	1.17	(0.84,1.65)	0.35	0.01	0.11	− 0.20	0.22	0.94
JIA								
Low maternal education	1.06	(0.43, 2.62)	0.90	− 0.30	0.29	− 0.87	0.26	0.30
Middle maternal education	1.31	(0.80, 2.16)	0.28	0.03	0.16	− 0.28	0.34	0.84
Low income	1.59	(0.81, 3.16)	0.18	0.17	0.22	− 0.26	0.60	0.44
Middle income	1.17	(0.65, 2.12)	0.60	− 0.01	0.19	− 0.38	0.36	0.95
Ulcerative colitis								
Low maternal education	1.64	(0.76, 3.51)	0.21	-0.19	0.14	− 0.47	0.09	0.18
Middle maternal education	1.15	(0.69, 1.90)	0.59	0.00	0.10	− 0.19	0.20	0.99
Low income	0.83	(0.42, 1.66)	0.60	− 0.12	0.13	− 0.38	0.13	0.34
Middle income	0.86	(0.50, 1.49)	0.61	− 0.05	0.11	− 0.26	0.15	0.61
Crohn’s disease								
Low maternal education	0.96	(0.36, 2.56)	0.93	− 0.09	0.28	− 0.63	0.45	0.75
Middle maternal education	0.91	(0.52, 1.58)	0.74	− 0.19	0.14	− 0.47	0.09	0.18
Low income	0.58	(0.24, 1.36)	0.21	0.04	0.21	− 0.37	0.45	0.86
Middle income	0.81	(0.45, 1.48)	0.50	0.05	0.15	− 0.24	0.34	0.74
Thyroid disease								
Low maternal education	1.10	(0.40, 2.98)	0.86	0.13	0.10	− 0.07	0.33	0.22
Middle maternal education	0.97	(0.55, 1.72)	0.91	− 0.03	0.06	− 0.15	0.08	0.61
Low income	1.38	(0.57, 3.33)	0.48	− 0.05	0.09	− 0.13	0.23	0.57
Middle income	1.33	(0.64, 2.76)	0.44	0.09	0.08	− 0.06	0.24	0.23
AD								
Low maternal education	1.29	(0.96, 1.73)	0.09	− 0.04	0.09	− 0.21	0.14	0.70
Middle maternal education	1.21	(1.01, 1.44)	0.04	− 0.02	0.05	− 0.13	0.09	0.71
Low income	0.96	(0.74, 1.24)	0.75	0.02	0.08	− 0.14	0.17	0.81
Middle income	1.08	(0.88, 1.32)	0.45	0.01	0.06	− 0.11	0.14	0.82
AD excluding Type 1 Diabetes								
Low maternal education	1.14	(0.81, 1.60)	0.46	− 0.07	0.11	− 0.28	0.14	0.52
Middle maternal education	1.11	(0.91, 1.35)	0.32	− 0.01	0.06	− 0.13	0.11	0.87
Low income	0.94	(0.70, 1.27)	0.70	0.01	0.09	− 0.17	0.19	0.91
Middle income	1.09	(0.87, 1.37)	0.46	0.00	0.07	− 0.14	0.14	0.98

‘Time to’ assess the association between SES and number of healthy days before developing an autoimmune disease. Complete cases: Maternal Education N 15,712, Household income N 15,698.

Multivariate model adjusted for child’s sex, ethnicity, and heredity of autoimmune disease, high maternal education/income was used as reference category ie RR 1.00. *AD* Autoimmune Disease, *JIA* Juvenil Idiopathic Arthritis. Coefficients for the “Time to disease diagnosis” model should be interpreted as positive indicating longer time-to-onset for the duration component. For education and income, “high” acts as the reference category. All RR values estimate the relative risk in that category relative to the high category.

There was no association between SES and time-to-onset of any autoimmune diseases, see Table 2. However, given the small number of individuals who developed individual autoimmune diseases this component generally has low statistical power.

Results for potential mediators of the association between SES—Type 1 Diabetes showed that three variables investigated had a statistically significant association with Type 1 Diabetes, these were BMI at 5 years of age, SLE at 5 years of age, and Infections during gestational month 3, see Supplementary Table 2. Out of these variables, two (BMI and SLE) were more common in low SES groups and were included in a path model to examine their role in the SES—Type 1 Diabetes association. Infections during gestational month 3 was reported more frequently by mother with high education and thus did not full fil the criteria of a role in low SES—Type 1 Diabetes association and was excluded.

**Figure 1:**
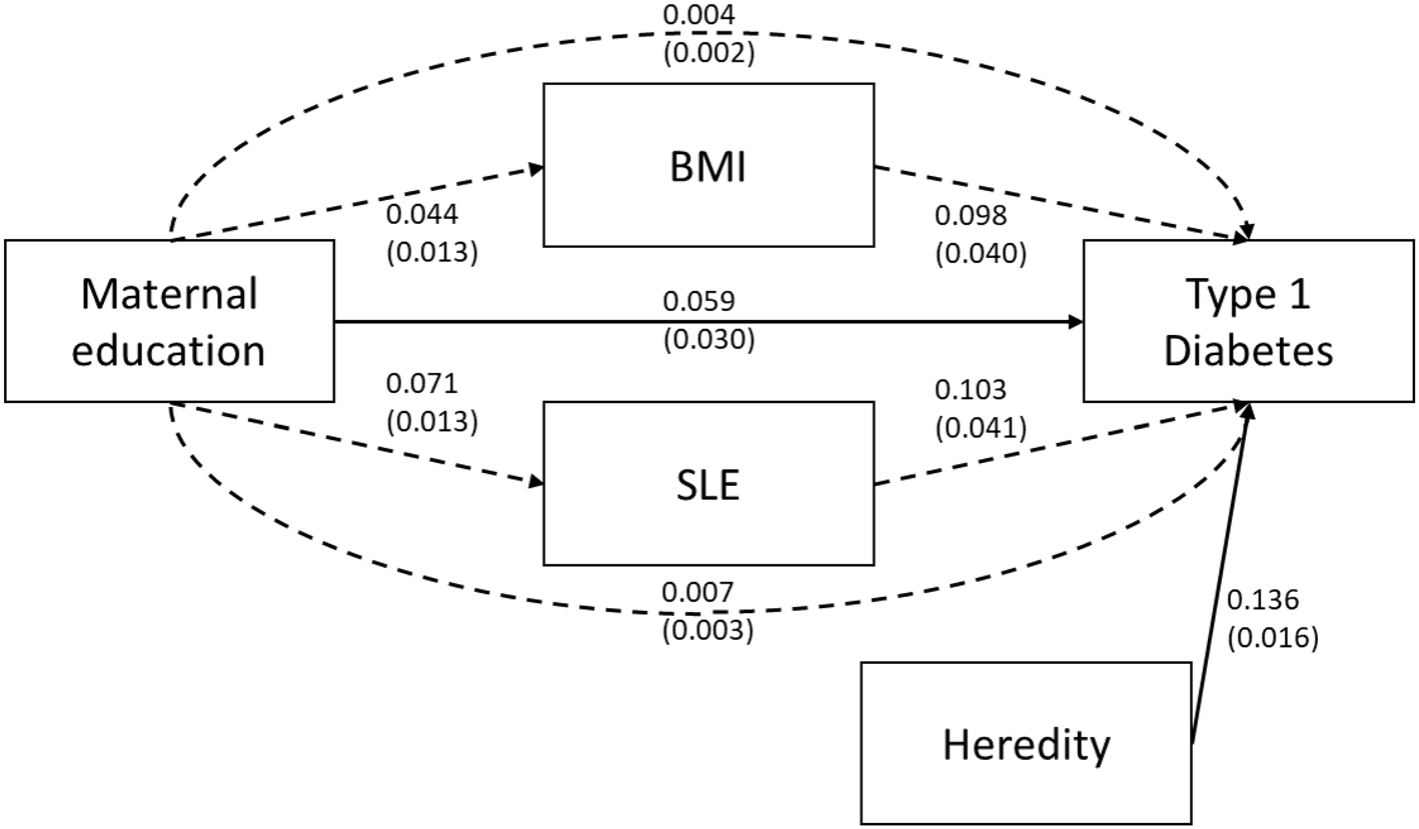
Mediation model of pathways between Maternal Education and Type 1 Diabetes. Direct pathway between maternal education and Type 1 Diabetes (from age 5 to 22, Type 1 Diabetes N 56, Complete cases N 6782) and indirect pathways through body mass index and serious life events at 5 years of age, adjusted for Heredity of Diabetes. Solid lines indicate direct paths while dashed lines indicate indirect paths. All path coefficients are reported in standardized form with standard errors in parentheses. The model’s overall fit was good with P = 0.02 in comparing the model in figure with the saturated model, RMSEA = 0.012 and CFI = 0.934. All fit metrics indicate good fit except for the comparison with the saturated model, however with such a large sample size the power of this test for small deviation is very high.

Path modelling included 56 participants with Type 1 Diabetes diagnosis after age 5 with complete information on all variables (maternal education, Type 1 Diabetes diagnosis, heredity of Type 1 Diabetes and BMI and SLE at age 5), N 6782. The analysis showed that out of the total effect of Low maternal education on Type 1 Diabetes (0.071 SE 0.029 P = 0.016) a small but statistically significant part was mediated through the two indirect pathways via BMI and SLE (0.012 SE 0.004 P = 0.003) which represents 17% of the total association. The mediating pathways were of equal effect size and largely independent of each other, BMI at age 5 (0.004 SE 0.002 P = 0.055) and SLE at age 5 (0.007 SE = 0.003 P = 0.025). Model fit was evaluated via the Chi-Square test comparing the model represented in Fig. 1 with the saturated (perfect) model where P > 0.05 is ideal, the root mean square error of approximation (RMSEA) where < 0.05 is ideal and the comparative fit index (CFI) where > 0.9 is ideal^25^.

## Discussion

In this Swedish prospective cohort study, we found a SES gradient in Type 1 Diabetes when SES was measured by maternal education. For the other investigated autoimmune diseases (Celiac Disease, Ulcerative colitis, Crohn’s Disease, JIA, Autoimmune hypo- and hyperthyroidism) no differences were detected between the three educational levels. There was no association between income and any of the autoimmune diseases, including Type 1 Diabetes.

Type 1 Diabetes differs from other autoimmune disease by being equally common in males and females during childhood, and twice as common in males after the age of 15 years^26,27^. Type 1 Diabetes also has a strong association with overweight and obesity while the association with other autoimmune diseases is less clear although some studies indicates that obesity increases the risk of MS, SLE, RA, IBD and Psoriasis^28^, while the risk of some autoimmune diseases including Celiac disease is decreased in obese individuals^29^. In children, obesity seems to increase the risk of Type 1 Diabetes and IBD more than JIA and Hypothyroidism^30^. The theoretical explanation for the association between Type 1 Diabetes and higher BMI could be the importance of beta-cell stress in disease development of Type 1 Diabetes. Insulin demand is increased as BMI increases and according to the “accelerator hypothesis” high insulin demand could accelerate beta-cell destruction by increasing the metabolic demand of beta-cells^31,32^. Higher BMI also interact directly with the autoimmune process and increases the risk of developing autoimmune antibodies in children > 9 years^33^. Psychosocial stress, such as serious life events, which has been shown to increase the risk of Type 1 Diabetes, could act either by increasing insulin demand as stress releases hormones including adrenalin and cortisol that increases blood glucose levels, or by influencing the immune balance^21^. The special role of beta cell stress in Type 1 Diabetes development could thus be part of the explanation why Type 1 Diabetes has a SES gradient, which other autoimmune diseases lacks. This hypothesis is strengthened by the mediating effect of BMI and SLE on the maternal education—Type 1 Diabetes association found in our study.

Autoimmune disease is more common in high income countries, and there is also a geographical difference with a north-west/south-east gradient with higher prevalence in countries in the north-west^34^. This has led to hypotheses that western lifestyle including dietary changes could explain differences in autoimmune prevalence. Our study indicates that several autoimmune diseases do not show any clear SES gradient in contrast to other disease groups influenced by western diet such as cardiovascular disease^35,36^. The lack of evidence for a SES gradient in most autoimmune diseases except Type 1 Diabetes in our and other studies suggests that the environmental factors responsible for the increase in autoimmune diseases should not mainly be sought among factors associated with low SES in western populations such as low physical activity, unhealthy diet or smoking^37^. Rather other environmental factors or life-style changes that affect the whole population more equally such as epidemic infections^38^, environmental toxins and pollutions^13^, and dietary changes like food additives and processing^39^ are examples of factors that should be targets of investigation. Other potential factors could be the increasing hygiene^40^, changes of microbiome of the body such as skin, respiratory tract or gut microbiome^41^.

We have found in repeated studies that in the Swedish ABIS cohort, in contrast to other birth cohorts conducted in high-income countries, the association between health outcomes and income is weaker than the association with maternal education^42–44^. Income differences between families with children in early childhood is reduced in Sweden due to policies including the parental leave insurance that have an upper level of compensation and by the fact that high educated fathers take longer parental leave^43,45^. Our conclusion is that maternal education is the preferred measure of childhood SES in the Swedish context and better reflect the status or social position of parents than household income. It is therefore not surprising that we found no association between income and Type 1 Diabetes in this study.

### Strength and limitations

This is a large prospective cohort study with more than 22 years of follow-up. The use of register data for the outcome variables made the loss to follow-up minimal. Still, the power to find significant differences for time-to-diagnosis is rather low because this analysis only include subject that developed each disease. Autoimmune diseases together affect only a few percent of the population and some of these diseases have a peak incidence in older age than our study subjects have yet reached. However, for the calculation of relative risks, our study had adequate power.

The effect size of the mediating effect of BMI on the risk of Type 1 Diabetes should be interpreted with caution. BMI was only measured at one timepoint and the effect could be larger in older children and adolescents because SES differences in BMI increases with age^46^. The association between BMI and Type 1 Diabetes autoantibody development is also stronger in higher age categories^33^. Another limitation of the mediation analysis is the loss to follow-up that resulted in only 56 out of 167 children with Type 1 Diabetes having complete data on variables necessary for this part of the study.

That high SES mothers reported increased prevalence of infections during early pregnancy should also be interpreted with caution. These data were collected when the child was born, and thus there is a risk of recall bias. Research have shown that recall bias is larger in low SES participants, which could explain the found differences^47^.

### Generalizability

The ABIS population is representative to the Swedish population in terms of percentage of educational groups^48^. One possibility for the lack of a SES gradient in autoimmune disease except Type 1 Diabetes found in this study could be due the choice of study country. However, previous studies on child health in ABIS have shown marked socioeconomic inequality with other outcomes such as ADHD, cardiovascular risk factor and infectious disease which makes this explanation less likely^44,49,50^. Even so, the SES gradient of Type 1 Diabetes and the lack of association between SES and other autoimmune diseases needs to be studied in less egalitarian countries, as differences in socioeconomic environment could influence the relationship between SES and disease.

## Conclusions

The risk of Type 1 Diabetes showed a gradient by maternal education, being increasingly higher with lower educational level. No association with maternal education or income was found for other investigated autoimmune diseases (Celiac Disease, Ulcerative colitis, Crohn’s Disease, JIA, Autoimmune hypo- and hyperthyroidism). Higher BMI and higher risk of serious life events mediated part of the increased risk in children to mothers with low education. The differences between Type 1 Diabetes and other autoimmune diseases could potentially be due the importance of insulin demand and beta-cell stress in Type 1 Diabetes development.

## Supplementary Information

Supplementary Tables.

## Data Availability

Data from the ABIS registry, including study protocols, report forms and consents may be obtained through requests to the PI and project coordinator Senior professor Johnny Ludvigsson email: Johnny.Ludvigsson@liu.se.
