# Supplementary material for: Low maternal education increases the risk of Type 1 Diabetes, but not other autoimmune diseases: a mediating role of childhood BMI and exposure to serious life events
Source: Sci Rep. 2023 Apr 15;13:6166. doi: 10.1038/s41598-023-32869-x (PMC10105777; doi:10.1038/s41598-023-32869-x)
Supplement: Supplementary file 1 — Supplementary Tables. [file 41598_2023_32869_MOESM1_ESM.docx]

| *Supplementary table 1. Multivariate analysis with both SES measures analyzed combined and with RRs for confounding variables, and the relative risk of autoimmune diseases. ‘Time to’ assess the association between SES and number of healthy days before developing an autoimmune disease.* | | | | | | | | | |
| --- | --- | --- | --- | --- | --- | --- | --- | --- | --- |
|  |  | Relative Risk of Disease | | | Time to disease diagnosis | | | | |
|  |  | RR | CI | p | b | SE | Lower | Upper | p |
| **Type 1 diabetes** | Low maternal education | 1.90 | (1.09, 3.30) | 0.02 | 0.10 | 0.18 | -0.25 | 0.45 | 0.563 |
|  | Middle maternal education | 1.56 | (1.07, 2.27) | 0.02 | -0.05 | 0.11 | -0.27 | 0.17 | 0.665 |
|  | Low income | 0.83 | (0.51, 1.37) | 0.47 | 0.09 | 0.16 | -0.23 | 0.41 | 0.588 |
|  | Middle income | 0.97 | (0.65, 1.44) | 0.87 | 0.13 | 0.12 | -0.10 | 0.37 | 0.274 |
|  | Heredity of T1D | 7.47 | (5.01, 11.14) | <0.01 | -0.16 | 0.12 | -0.40 | 0.08 | 0.188 |
|  | Child’s sex: female | 0.88 | (0.65, 1.20) | 0.43 | -0.08 | 0.09 | -0.26 | 0.10 | 0.383 |
|  | Ethnicity: immigrant parent | 0.45 | (0.22, 0.92) | 0.03 | 0.02 | 0.22 | -0.41 | 0.44 | 0.938 |
| **Celiac disease** | Low maternal education | 1.04 | (0.61, 1.78) | 0.89 | -0.23 | 0.17 | -0.56 | 0.11 | 0.190 |
|  | Middle maternal education | 1.10 | (0.82, 1.49) | 0.52 | 0.05 | 0.09 | -0.14 | 0.23 | 0.624 |
|  | Low income | 0.83 | (0.52, 1.32) | 0.43 | 0.03 | 0.15 | -0.26 | 0.32 | 0.850 |
|  | Middle income | 1.14 | (0.81, 1.62) | 0.46 | 0.01 | 0.11 | -0.20 | 0.22 | 0.912 |
|  | Heredity of CD | 5.34 | (3.21, 8.87) | <0.01 | -0.17 | 0.17 | -0.49 | 0.16 | 0.312 |
|  | Child’s sex: female | 2.03 | (1.54, 2.67) | <0.01 | 0.10 | 0.09 | -0.07 | 0.27 | 0.268 |
|  | Ethnicity: immigrant parent | 0.30 | (0.14, 0.63) | <0.01 | 0.18 | 0.23 | -0.28 | 0.64 | 0.433 |
| **JIA** | Low maternal education | 0.96 | (0.39, 2.37) | 0.93 | -0.36 | 0.30 | -0.94 | 0.23 | 0.229 |
|  | Middle maternal education | 1.25 | (0.76, 2.05) | 0.37 | 0.01 | 0.16 | -0.32 | 0.33 | 0.970 |
|  | Low income | 1.53 | (0.77, 3.03) | 0.22 | 0.21 | 0.23 | -0.24 | 0.67 | 0.359 |
|  | Middle income | 1.12 | (0.62, 2.02) | 0.71 | 0.02 | 0.20 | -0.37 | 0.40 | 0.938 |
|  | Heredity of JIA | 1.60 | (0.39, 6.47) | 0.51 | 0.30 | 0.46 | -0.59 | 1.20 | 0.509 |
|  | Child’s sex: female | 2.32 | (1.46, 3.69) | <0.01 | 0.19 | 0.15 | -0.10 | 0.49 | 0.195 |
|  | Ethnicity: immigrant parent | 0.51 | (0.21, 1.25) | 0.14 | -0.13 | 0.31 | -0.73 | 0.48 | 0.680 |
| **Ulcerative colitis** | Low maternal education | 1.74 | (0.79, 3.80) | 0.17 | -0.17 | 0.15 | -0.46 | 0.11 | 0.237 |
|  | Middle maternal education | 1.20 | (0.71, 2.02) | 0.51 | 0.01 | 0.10 | -0.19 | 0.20 | 0.936 |
|  | Low income | 0.76 | (0.37, 1.55) | 0.45 | -0.10 | 0.13 | -0.35 | 0.17 | 0.479 |
|  | Middle income | 0.83 | (0.47, 1.45) | 0.51 | -0.05 | 0.11 | -0.26 | 0.15 | 0.616 |
|  | Heredity of IBD | 4.58 | (1.87, 11.20) | <0.01 | -0.05 | 0.18 | -0.41 | 0.31 | 0.768 |
|  | Child’s sex: female | 1.17 | (0.75, 1.82) | 0.49 | 0.00 | 0.09 | -0.18 | 0.18 | 1.000 |
|  | Ethnicity: immigrant parent | 1.37 | (0.71, 2.63) | 0.35 | -0.14 | 0.12 | -0.38 | 0.10 | 0.261 |
| **Crohn’s disease** | Low maternal education | 1.08 | (0.38, 3.03) | 0.89 | -0.07 | 0.29 | -0.65 | 0.50 | 0.807 |
|  | Middle maternal education | 0.97 | (0.54, 1.74) | 0.92 | -0.20 | 0.14 | -0.48 | 0.08 | 0.172 |
|  | Low income | 0.58 | (0.24, 1.43) | 0.24 | 0.07 | 0.21 | -0.33 | 0.48 | 0.724 |
|  | Middle income | 0.82 | (0.43, 1.56) | 0.54 | 0.05 | 0.15 | -0.25 | 0.35 | 0.730 |
|  | Heredity of IBD | 4.71 | (1.75, 12.71) | <0.01 | -0.11 | 0.31 | -0.71 | 0.50 | 0.732 |
|  | Child’s sex: female | 0.79 | (0.47, 1.33) | 0.38 | 0.22 | 0.14 | -0.04 | 0.49 | 0.103 |
|  | Ethnicity: immigrant parent | 0.67 | (0.24, 1.87) | 0.44 | 0.00 | 0.25 | -0.49 | 0.49 | 0.999 |
| **Thyroid disease** | Low maternal education | 1.03 | (0.36, 2.93) | 0.96 | 0.12 | 0.10 | -0.08 | 0.32 | 0.238 |
|  | Middle maternal education | 0.92 | (0.51, 1.64) | 0.77 | -0.05 | 0.06 | -0.17 | 0.06 | 0.374 |
|  | Low income | 1.40 | (0.56, 3.53) | 0.47 | -0.08 | 0.09 | -0.10 | 0.25 | 0.386 |
|  | Middle income | 1.36 | (0.65, 2.84) | 0.42 | 0.12 | 0.08 | -0.03 | 0.26 | 0.129 |
|  | Heredity of AT | 3.96 | (1.59, 9.84) | <0.01 | -0.11 | 0.10 | -0.31 | 0.10 | 0.305 |
|  | Child’s sex: female | 4.34 | (1.59, 9.84) | <0.01 | 0.04 | 0.07 | -0.09 | 0.18 | 0.533 |
|  | Ethnicity: immigrant parent | 0.48 | (0.15, 1.56) | 0.22 | -0.11 | 0.12 | -0.34 | 0.12 | 0.343 |
| **AD** | Low maternal education | 1.32 | (0.98, 1.78) | 0.06 | -0.04 | 0.09 | -0.22 | 0.14 | 0.661 |
|  | Middle maternal education | 1.21 | (1.01, 1.46) | 0.04 | -0.02 | 0.06 | -0.13 | 0.09 | 0.668 |
|  | Low income | 0.90 | (0.69, 1.17) | 0.42 | 0.03 | 0.08 | -0.13 | 0.19 | 0.726 |
|  | Middle income | 1.03 | (0.84, 1.27) | 0.78 | 0.02 | 0.06 | -0.10 | 0.14 | 0.762 |
|  | Heredity of AD | 2.27 | (1.86, 2.78) | <0.01 | -0.08 | 0.06 | -0.20 | 0.05 | 0.234 |
|  | Child’s sex: female | 1.48 | (1.27, 1.74) | <0.01 | 0.03 | 0.05 | -0.07 | 0.13 | 0.544 |
|  | Ethnicity: immigrant parent | 0.56 | (0.40, 0.78) | <0.01 | 0.06 | -0.10 | -0.14 | 0.25 | 0.578 |
| Multivariate model adjusted for child’s sex, ethnicity, and heredity of autoimmune disease. AD: Autoimmune Disease, AT: Autoimmune Thyroid disease (Graves’ Disease or Hashimoto’s Disease), IBD: inflammatory bowel disease (Crohn’s Disease or Ulcerative Colitis), JIA: Juvenil Idiopathic Arthritis, T1D: Type 1 Diabetes. Coefficients for the “Time to disease diagnosis” model should be interpreted as positive indicating longer time-to-onset for the duration component. | | | | | | | | | |

| *Supplementary table 2. Distribution of potential mediators by maternal education and mediator - Type 1 Diabetes association.* | | | | | | | | | |
| --- | --- | --- | --- | --- | --- | --- | --- | --- | --- |
|  |  | Maternal Education | | | | | Type 1 Diabetes | | |
|  |  | Total | High | Middle | Low |  |  | Relative Risk |  |
|  |  | N (%)  or M(SD) | N (%)  or M(SD) | N (%)  or M(SD) | N (%)  or M(SD) | p-value^1^ | RR | CI | p-value^2^ |
| **Infection GA 3 months** | |  |  |  |  |  |  |  |  |
|  | Yes | 385 (2.4) | 182 (3.6) | 183 (1.9) | 20 (1.5) | <0.001 | 3.20 | (1.80, 5.72) | <0.001 |
|  | No | 15587 (97.6) | 4886 (96.4) | 9342 (98.1) | 1359 (98.5) |  | Ref |  |  |
| **LGA** | |  |  |  |  |  |  |  |  |
|  | Yes | 743 (4.8) | 231 (4.6) | 437 (4.7) | 75 (5.7) | 0.232 | 0.92 | (0.43, 1.95) | 0.827 |
|  | No | 14807 (95.2) | 4741 (95.4) | 8834 (95.3) | 1232 (94.3) |  | Ref |  |  |
| **SLE at age 5** | |  |  |  |  |  |  |  |  |
|  | Yes | 1541 (22.0) | 469 (19.2) | 932 (22.6) | 140 (31.8) | <0.001 | 1.98 | (1.25, 3.14) | 0.004 |
|  | No | 5475 (78.0) | 1975 (80.8) | 3200 (77.4) | 300 (68.2) |  | Ref |  |  |
| **Weight group mother** | |  |  |  |  |  |  |  |  |
|  | Obese | 739 (7.2) | 153 (4.5) | 494 (8.1) | 92 (13.0) | <0.001 | 1.02 | (0.47, 2.22) | 0.957 |
|  | Overweight | 2327 (22.8) | 652 (19.0) | 1483 (24.4) | 192 (27.0) |  | 1.31 | (0.84, 2.03) | 0.232 |
|  | Normal | 7152 (70.0) | 2633 (76.6) | 4093 (67.4) | 426 (60.0) |  | Ref |  |  |
| **BMI Child at age 5** | |  |  |  |  |  |  |  |  |
|  | BMI | 16.04 (1.68) | 15.94 (1.54) | 16.10 (1.73) | 16.11 (2.00) | 0.001 | 1.18 | (1.06, 1.32) | 0.002 |
| BMI; Body Mass Index, GA; Gestational Age, LGA; Large for Gestational Age, SLE; Serious Life Events.  ^1^ P-values are calculated by χ^2^ for categorical variables and by ANOVA for continuous variable (BMI).  ^2^ P-values by poison regression using robust variance estimation. | | | | | | | | | |
